# Defining the South African Acute Respiratory Infectious Disease Season

**DOI:** 10.3390/ijerph20021074

**Published:** 2023-01-07

**Authors:** Ogone Motlogeloa, Jennifer M. Fitchett, Neville Sweijd

**Affiliations:** 1 School of Geography, Archaeology and Environmental Studies, University of the Witwatersrand, Johannesburg 2050, South Africa; 2 Alliance for Collaboration on Climate and Earth Systems Science (ACCESS), Council for Scientific and Industrial Research (CSIR), Pretoria 0001, South Africa

**Keywords:** influenza, common cold, RSV, seasonality, winter, public health

## Abstract

The acute respiratory infectious disease season, or colloquially the “flu season”, is defined as the annually recurring period characterized by the prevalence of an outbreak of acute respiratory infectious diseases. It has been widely agreed that this season spans the winter period globally, but the precise timing or intensity of the season onset in South Africa is not well defined. This limits the efficacy of the public health sector to vaccinate for influenza timeously and for health facilities to synchronize efficiently for an increase in cases. This study explores the statistical intensity thresholds in defining this season to determine the start and finish date of the acute respiratory infectious disease season in South Africa. Two sets of data were utilized: public-sector hospitalization data that included laboratory-tested RSV and influenza cases and private-sector medical insurance claims under ICD 10 codes J111, J118, J110, and J00. Using the intensity threshold methodology proposed by the US CDC in 2017, various thresholds were tested for alignment with the nineteen-week flu season as proposed by the South African NICD. This resulted in varying thresholds for each province. The respiratory disease season commences in May and ends in September. These findings were seen in hospitalization cases and medical insurance claim cases, particularly with influenza-positive cases in Baragwanath hospital for the year 2019. These statistically determined intensity thresholds and timing of the acute respiratory infectious disease season allow for improved surveillance and preparedness among the public and private healthcare.

## 1. Introduction

Acute respiratory diseases broadly refer to any infectious diseases of the upper or lower respiratory tract [1]. The body’s respiratory system includes the nose, sinuses, mouth, throat (pharynx), voice box (larynx), windpipe (trachea), and lungs [1]. Upper respiratory infections affect the nose, sinuses, and throat, while lower respiratory infections affect the airways and lungs [1,2]. Influenza and respiratory syncytial virus (RSV) infections are common causes of lower respiratory tract infections [3,4,5]. Although not all instances of influenza infection seek medical attention and are thus not all detected by the surveillance systems, the annual rate of influenza infection is estimated to be between 5 and 10% in adults and between 20 and 30% in children during a typical season [6].

Studies reveal that the burden of influenza is particularly high in sub-Saharan Africa, particularly in South Africa, where influenza-associated mortality rates are greater than in other areas [7]. Between 7000 and 12,000 seasonal influenza-related deaths are estimated to occur annually in South Africa [7]. It is generally agreed that the following goals should be covered by influenza surveillance: keeping track of the circulating viral strains, the frequency, intensity, and severity of epidemic waves; letting people know about the risk factors that contribute to severity; and providing epidemiological and virological assistance for pandemic early warning and readiness [8]. Indicators, both quantitative and qualitative, that measure the impact of seasonal outbreaks are used to support influenza surveillance [8,9]. Clinical consultations in general practice, hospitalized laboratory-confirmed cases, sentinel and non-sentinel positive specimens, mortality, and local epidemics are the main sources of these indicators [10]. The estimated incidence of the percentage of consultations in a population in a certain period, which is connected to the severity of seasonal epidemics, is one of the most crucial indicators in influenza surveillance. For analyzing the dynamics of influenza epidemics and comparing patterns within or across countries, overall seasonal or weekly influenza intensity levels are helpful [11]. It is important to maintain a comprehensive system for influenza surveillance for the following reasons: (1) vaccines must be administered annually and are updated regularly based on surveillance findings, (2) the need to inform the public to be cautious, and (3) the need for hospital medical practice readiness [10]. There is increasing evidence that suggests that the length of a single flu season on regional scales has changed under global warming, but a hemispherical-scale response to the four seasons in the past and future remains unknown [12]. Therefore, influenza surveillance needs to take into account the climate change effect of these trends in the seasonal timing and intensity of this disease [12,13,14].

There is a broad consensus that in countries with distinct climatic seasonality, there is an increase in the incidence of acute infectious respiratory disease caseload during winter as meteorological conditions are more suitable for the survival of acute infectious respiratory viruses [10,15,16]. Despite a widespread understanding of this winter seasonality in acute infectious respiratory diseases, there is little standardization in how to define the onset or timing of the season. The acute respiratory infectious disease season is seldom quantified, and when it is, there is no standardization in the quantification methodologies.

In 2017, the Centers for Disease Control and Prevention (CDC) outlined and adopted a new methodology for classifying flu season severity [17]. Based on data from past flu seasons, the CDC used key flu indicator data to develop intensity thresholds (ITs) to classify the severity of flu seasons and set a specific threshold to delineate the start of the influenza season [17,18]. An intensity threshold is a value developed using data from past flu seasons that help assess the probability that an influenza indicator, such as influenza-like-illness (ILI) or influenza percent positive, will go above a certain threshold [17,18]. The National Institute of Communicable Diseases (NICD) in South Africa states that the average duration of the influenza season over the past thirteen years has been nineteen weeks [7] but has not set an intensity threshold nor published any methodologies for the delineation of the influenza season. Globally, the definitions for the acute respiratory infectious disease season and its onset thresholds vary. These include regions that refer to the season being defined by a ‘significant spike’ but do not indicate how that is quantified. Other regions define the season by the month of the largest number of cases, but in these instances one can only define the timing of the season retrospectively, affording little value in adapting the health sector response through the season [17,19]. Where thresholds have been used, they vary widely. Eurich et al. [20] argue that the respiratory disease season commences from the first case and ends with the last case in the year, whilst a European study advocates for a 5% threshold. Using a range of data sets from the public and private healthcare sectors in South Africa, this study applies the CDC [17] method of intensity thresholds, combined with the NICD stipulated length of the influenza season to explore the timing of onset and cessation of the acute infectious respiratory disease season and the relevant thresholds across the region.

## 2. Materials and Methods

### 2.1. Study Site

**Figure 1:**
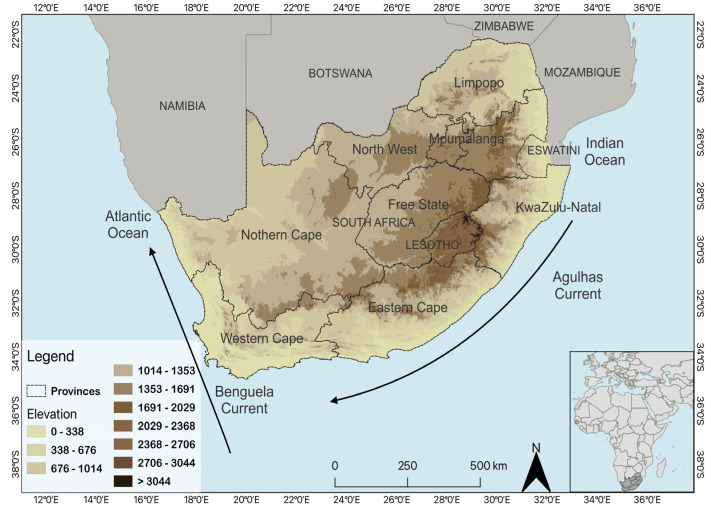
Map of the study site of South Africa indicating the nine provinces, topography, the surrounding oceans, and their respective currents.

The southernmost country in Africa, South Africa, has 9 provinces and a land area of 1,220,000 km^2^. It is situated between the latitudes of 22–35° S and 16–33° E. (Figure 1) [21,22,23]. There is considerable climatic heterogeneity across the country: hot, dry, and arid conditions in the west and warm, wet, and humid conditions in the east [21,23,24]. Rainfall occurs in summer across much of the country, but the southwestern tip is characterized by winter rainfall, and the southern coast by year-round rainfall (Figure 1) [25,26,27,28,29].

The National Department of Health (NDoH) in South Africa is in charge of overseeing, coordinating, and setting policies for the nation’s health services, while the nine provincial departments are primarily in charge of providing those services [30]. A two-tiered, severely unequal healthcare system exists in South Africa. The majority of the population (71%), which is served by the public sector, is funded by the government [30,31]. Around 27% of the population is served by the private sector, which is mostly financed by individual payments to health insurance or medical insurance plans [30,31,32,33].

### 2.2. Data Sources

Acute respiratory diseases, in the context of this paper, are defined as infections of the respiratory system caused by viruses or bacteria that develop in less than fifteen days and show symptoms such as coughing, nasal congestion, airway obstruction, sore throat, dysphonia, or respiratory distress, whether they are accompanied by fever [1]. The conditions under this wide classification that are the subject of this study include influenza, respiratory syncytial virus (RSV), acute nasopharyngitis, influenza with pneumonia, influenza with other respiratory manifestations, and influenza with other features. The most frequent reasons for primary care visits are influenza and other respiratory viral illnesses, which place a significant financial strain on the global economy [2,3,4,5].

To explore definitions for the start and finish of the South African acute respiratory infectious disease season, this study utilized two data sets. One data set encompassed a time series of five years of laboratory-tested and confirmed RSV and influenza hospitalization cases, and the other consisted of a time series of medical insurance scheme claims where claimants were diagnosed and treated symptomatically for acute respiratory disease. The two data sets are important for the following reasons: (1) from the magnitude of the medical insurance claims data, a seasonal temporal pattern can be ascertained; (2) the hospitalization cases—although a conservative estimate—indicate the actual number of extreme cases when people were hospitalized for an acute respiratory infectious disease. Although the data sets do not capture those cases that are self-diagnosed and treated with over-the-counter (OTC) medication; these two data sets are a sound index of incidence and enable us to compare hospitalizations cases and medical insurance claims which would be representative of the population levels of infection. It is important to note that both these data sets include data up to the end of 2019, specifically to avoid COVID-19 as it could potentially reflect on the medical insurance scheme claims under the ICD 10 code considering that these individuals are being treated symptomatically. Furthermore, it is yet to be proven that COVID-19 has a distinct seasonality in South Africa [13,34]. Ethical clearance to work with both data sets was obtained from the University of Witwatersrand Health Research Ethics Committee (HREC) with ethics clearance no: M210617 approved on 4 August 2021 and is valid up until 4 August 2026.

### 2.3. Hospitalization Cases

The hospitalization data utilized in this study were applied for and obtained through the South African Medical Research Council (SAMRC) Respiratory and Meningeal Pathogens Research Unit which has been conducting surveillance for respiratory viruses for over 20 years at Chris Hani Baragwanath Hospital. This research unit has recorded laboratory-confirmed influenza and RSV-associated hospitalization cases under ethics clearance no: 131109 through the Human Research Ethics Committee (HREC). Chris Hani Baragwanath Hospital is located in Soweto, a South African suburb southwest of central Johannesburg. It is Africa’s largest hospital and the world’s third-largest hospital, occupying 170 acres of land and housing 3400 beds and 6760 employees [35]. It is one of the 40 Gauteng provincial hospitals, and it is funded and managed by the Gauteng Provincial Department of Health [36]. It also serves as a teaching hospital for the University of the Witwatersrand Medical School [35,36]. Although these data were obtained from the largest hospital in Africa, its patients are still only a small proportion of all influenza and RSV cases in South Africa as it is only one hospital and accounts only for cases that are severe enough for hospitalization and laboratory testing to guide diagnosis. Therefore, these data are a conservative estimate of the pattern of the seasonality of hospitalization cases confirmed to be a result of an acute respiratory infectious disease.

### 2.4. Medical Insurance Scheme Claims

Discovery Medical Insurance Scheme is a private medical insurance company catering to 3.3 million clients across South Africa. The scheme retains all data on medical claims submitted to them, each of which is made against a specific ICD 10 code. These ICD 10 codes can relate to claims for visits with medical practitioners, any medical tests conducted, and medication dispensed for that condition. The medical practitioner bases the ICD 10 code listed for that consultation based on their diagnosis, but these do not require laboratory testing for confirmation. For this study, we obtained Discovery claims data for ICD 10 codes J00, J110, J111, and J118, which incorporate all cases of acute nasopharyngitis, influenza with pneumonia, influenza with other respiratory manifestations, and influenza with other manifestations. Data were provided as individual daily claims among all clients across the country from 2008 to 2019. Included in the metadata for each claim is the town in which the claim was made, the date of the claim, the age band the claimant belongs to, the claimant’s gender, and the number of doctor visits the claimant attended.

### 2.5. Data Analysis

To determine the acute respiratory infectious disease season in South Africa, various methods were employed. First, it was important to identify if there was a seasonality in medical insurance claims and hospitalization cases. In the interests of the comparability of the different data sets, the medical insurance claim data were converted to weekly totals, starting on Sunday of each week. These totals were then tabulated and plotted, commencing from the first week of the year 2008 to the last week of the year 2019. This process was repeated for all nine provinces from 2008 to 2019 to assess the seasonality of each province, respectively, given their distinct geographical location and climatologies, and independently for the Baragwanath hospitalization cases (Figure 1). This was proceeded by calculating and plotting a five-point running mean for each province. Exploratory data analysis was employed to determine whether there were any erroneous values in any of the data sets.

Once both data sets were represented graphically and a seasonality in claims and hospitalization cases were identified and confirmed, the start and end dates of the seasons were explored. While the NICD [7] states that the average duration of the influenza season over the past thirteen years has been nineteen weeks, no objective criteria have been established to determine the onset and cessation thresholds. We considered that internationally the acute respiratory infectious disease season has been defined through a variety of approaches. For example, Eurich et al. [20] argue that the respiratory disease season commences from the first case and ends with the last case in the year, whilst a European study [11] advocated for a 5% threshold. In light of this, we explored thresholds that resulted in the suggested nineteen weeks of acute respiratory disease season in South Africa.

To achieve this, we calculated the total number of cases for each year in each province and averaged those for the study period (2008–2019). The thresholds were calculated as percentages of the annual mean total number of cases for each province. When the percentage had been applied to the mean annual total for that province, a threshold value was returned. The weekly data were then coded as either exceeding this threshold or not and plotted accordingly. The start of the respiratory disease season was then defined as the week from which the threshold in cases is exceeded, and the end of the season is defined as the week from which the threshold is not exceeded. In the interests of comparability, we began by exploring the 5% threshold used for defining the onset and cessation of the influenza season in Europe [10]. We thereafter applied thresholds of 10%, 15%, 20%, 33.3%, 60%, and 90% to the data for each province to identify the threshold that returned a respiratory disease season that most closely aligned with the 19 weeks duration defined by the NICD [7]. The respiratory disease season defined by the respective threshold for each province was then also plotted as an indication of weeks that either exceeded or fell below that threshold.

## 3. Results

### 3.1. Weekly Trends in Hospitalization Cases and Medical Insurance Claims

**Figure 2:**
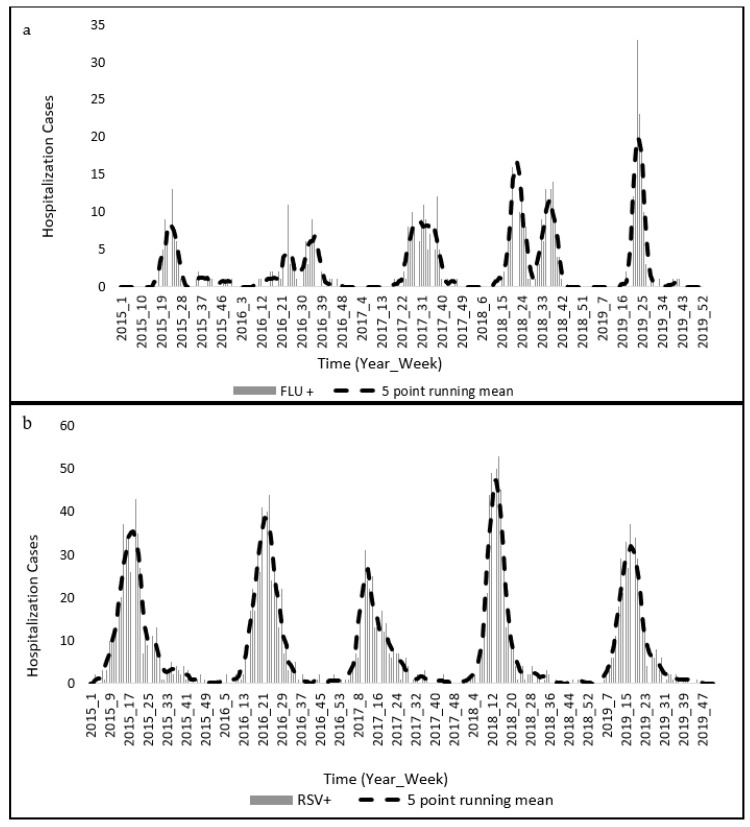
Caseloads of hospitalized patients with positive tests for (**a**) influenza and (**b**) RSV (2015–2019).

Influenza and RSV hospitalization rates at Baragwanath hospital indicate that cases peaked during early autumn to winter from 2015 to 2019 and influenza cases increased during winter in South Africa (Figure 2). A distinct single peak in cases per year is observed in RSV- positive cases and influenza-positive cases (Figure 2). However, these peaks are better observed in the RSV-positive cases in comparison to the influenza-positive cases (Figure 2). The RSV cases utilized in this study were obtained from the paediatric ward at Baragwanath hospital and show a distinct seasonality for the following reasons: (1) RSV is the most common cause of bronchitis and lower respiratory tract illness among young children, (2) infants and young children often have underdeveloped immune systems, and (3) transmissions often occur more frequently due to shared spaces such as schools and playgrounds [37]. Cases increase from close to 0 each week to over 7 a week for influenza and over 35 a week for RSV (Figure 2). Cases of influenza started to increase from week 20 in 2015, with the highest number of cases being during week 24 with a total of 13 hospitalizations (Figure 2a). In that same year, a spike in RSV is seen between weeks 8 and 9, 14 and 15 with the highest cases being observed in week 20 with a total of 43 cases (Figure 2b).

In 2016, influenza cases were relatively low. However, they spiked in week 24 with 11 cases and gradually increased again from week 31, and almost no cases were reported post-week 39 (Figure 2a). The prevalence of cases was observed for RSV from weeks 14 to 29, with the highest cases being observed in week 24 with 44 cases (Figure 2b). In 2017, RSV cases started to gradually increase from the first week, and during weeks 10 and 11, RSV cases were highest with a total of 31 cases for each, week respectively, and they gradually decreased after week 11 where almost no cases were reported from 30 until the remainder of the year (Figure 2b). Similarly, influenza case prevalence was low in 2017 with the highest number of cases being in week 38 with a total of 12 cases (Figure 2a). In 2018, cases started to increase from week 18, and week 22 reported the highest number of influenza cases which was 23, and RSV cases started to increase with the highest number of cases being observed in week 15 with a total of 53 cases after which it started to gradually decrease (Figure 2). Lastly, in 2019 influenza cases started to increase during week 21 and peaked during week 23 with 33 reported cases, and RSV cases started to increase during week 11 and peaked in week 17 with 37 cases (Figure 2). In 2019, there was a spike in influenza cases during week 23 that is not apparent for RSV (Figure 2). Overall, both influenza and RSV cases begin to gradually increase during the colder seasons and start to gradually decrease during the warmer seasons. However, in 2017 cases of influenza and RSV started to increase early, and overall, fewer people were hospitalized for both these two acute lower respiratory diseases.

RSV-positive hospitalized patients at Baragwanath hospital exceeded those who tested positive for influenza overall (Figure 2b). Across all the years, we were in the influenza season by week 21 and out of it roughly between weeks 37 and 40 and in the RSV season by week 13 and out of it roughly between weeks 32 and 34 (Figure 2). The peak in cases for influenza on average was experienced in weeks 21–25, and for RSV cases the peak was usually between weeks 17 and 23 (Figure 2). In 2015, RSV cases began to increase as early as week 9 and peaked at week 20 with a total of 43 cases. In 2016, RSV cases started to increase from week 15 and peaked at 44 cases in week 24. In 2017, cases exhibited similar patterns to those of 2015 and started increasing from week 6 and peaked in weeks 10 and 11 with 31 cases in each week. The number of cases per week increased in 2018. This increase started in week 7 and peaked in week 15 with 53 cases, and cases started to increase from week 11 in 2019 and peaked in week 19 with 34 cases.

**Figure 3:**
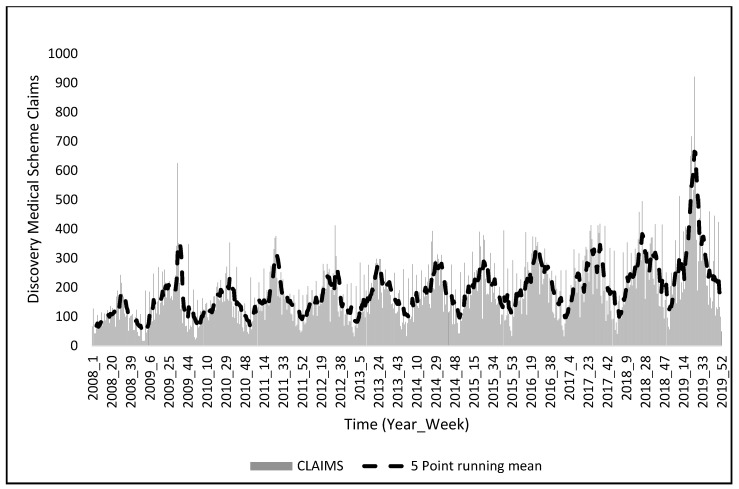
Weekly medical insurance claims for South Africa and the 5-point running mean (2008–2019).

Figure 3 is a graphical representation of the national claim totals for the Discovery medical insurance scheme. It is easy to deduce that there is a distinct seasonality in claims for the whole country. Nationwide claims peak in the colder seasons (autumn and winter) and decrease during the warmer seasons (spring and summer). There is an overall increase in claims from 2008 to 2019. In 2008, claims peaked at week 29. In 2009, claims peaked in week 34. In 2010, claims peaked in week 33. In 2011, claims peaked at week 25. In 2012, claims peaked at week 22, and in 2013, claims peaked in week 22. In 2014, claims peaked in week 26. In 2015, claims peaked in week 21. In 2016, claims peaked in week 21. In 2017, claims peaked in week 40. In 2018, claims peaked in week 25, and in 2019, claims peaked in week 22. It is clear that cases start to increase during colder seasons and decrease during the warmer seasons (Figure 3).

**Figure 4:**
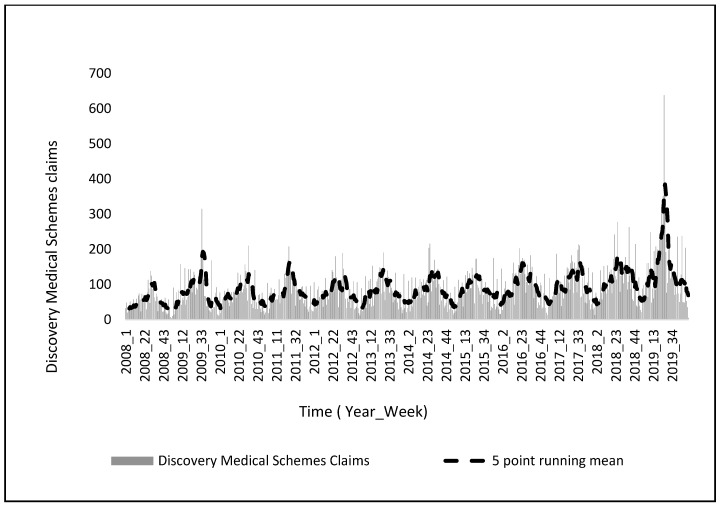
Discovery medical scheme claims in Gauteng, with a 5-point running, mean.

**Figure 5:**
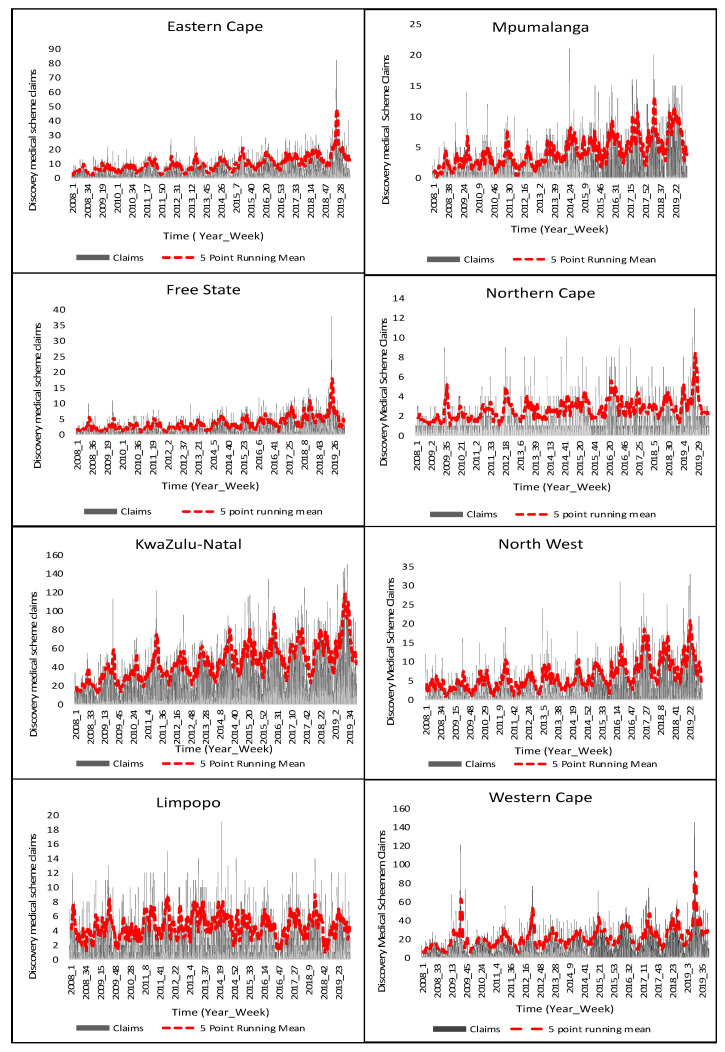
Medical insurance claims and running mean for Eastern Cape, Free State, KwaZulu-Natal, Limpopo, Mpumalanga, Northern Cape, North West Province, and Western Cape.

Exploring each province individually, a distinct seasonal pattern is observed in Gauteng province (Figure 4). It is the most densely populated province in South Africa, and it is also the economic hub of the country and consists of individuals that have more financial means and can subsequently afford medical insurance (Figure 4). Acute lower respiratory diseases spread mainly through droplets when infected people cough or sneeze making it a disease that spreads easily [17]. Given that the geographical space of Gauteng is the smallest in comparison to the other nine provinces, this subsequently means that there are more people per square kilometer. From 2015–2019, RSV or flu cases on average peaked in the winter season, roughly around week 21 (Figure 2). However, unlike Baragwanath hospitalization data, there are claims every week including the off-peak seasons which are the warmer seasons (Figure 4). However, like Baragwanath influenza cases, Discovery medical insurance claims in Gauteng were exceedingly higher in 2019 during the colder seasons (Figure 2). South Africa in general, experiences a climate that varies temporally and spatially across several scales, and this is because of its topography, surrounding oceans, and latitudinal location [26]. Therefore, not all nine provinces will exhibit a distinct seasonal pattern (Figure 5).

The Western Cape, Eastern Cape, and KwaZulu-Natal also show a distinct seasonality with the peak seasons being in the colder seasons and the off-peak seasons being the warmer seasons compared to the remainder of the other provinces (Figure 5). It is interesting to note that all three provinces are situated along the coast (Figure 1). However, the North West province exhibits a distinct seasonal pattern in comparison to the remaining provinces, which consist of the Free State, Limpopo, Mpumalanga, and the Northern Cape all situated inland and not exhibiting a distinct seasonality (Figure 1 and Figure 5).

Limpopo, the northernmost province of South Africa, exhibits no seasonal pattern in claims as the claims peaked in both peak and off-peak seasons from 2008 to 2019 (Figure 1 and Figure 5). This is also the case for the Northern Cape; however, in comparison to Gauteng province, many people cannot afford the luxury of health care insurance, and this province has the lowest number of claims (Figure 5). The Free State has a low number of claims similar to the influenza cases from Baragwanath hospital and from Gauteng. There was, however, a spike in claims during the winter season of 2019 (Figure 2a, Figure 4 and Figure 5). Mpumalanga also does not exhibit a distinct seasonality. A slight pattern can, however, be observed when assessing the five-point running mean graph (Figure 5).

### 3.2. Defining the Acute Infectious Respiratory Disease Season

Eurich et al. [20] argued that the respiratory disease season spans from the first case to the last case each year. This methodology would be effective for the Baragwanath hospitalization data for RSV and influenza. However, for the medical insurance claim data, there are claims for every week of the year in each of the provinces, and this method would return a respiratory disease season that spans the full 52 weeks of the year. As the first positive test for the hospitalization data is very unlikely to be the first person to contract an acute infectious respiratory disease in South Africa, the hospitalization data cannot be considered a clear indicator of disease prevalence in Gauteng province and definitely cannot be generalized to the full country.

**Figure 6:**
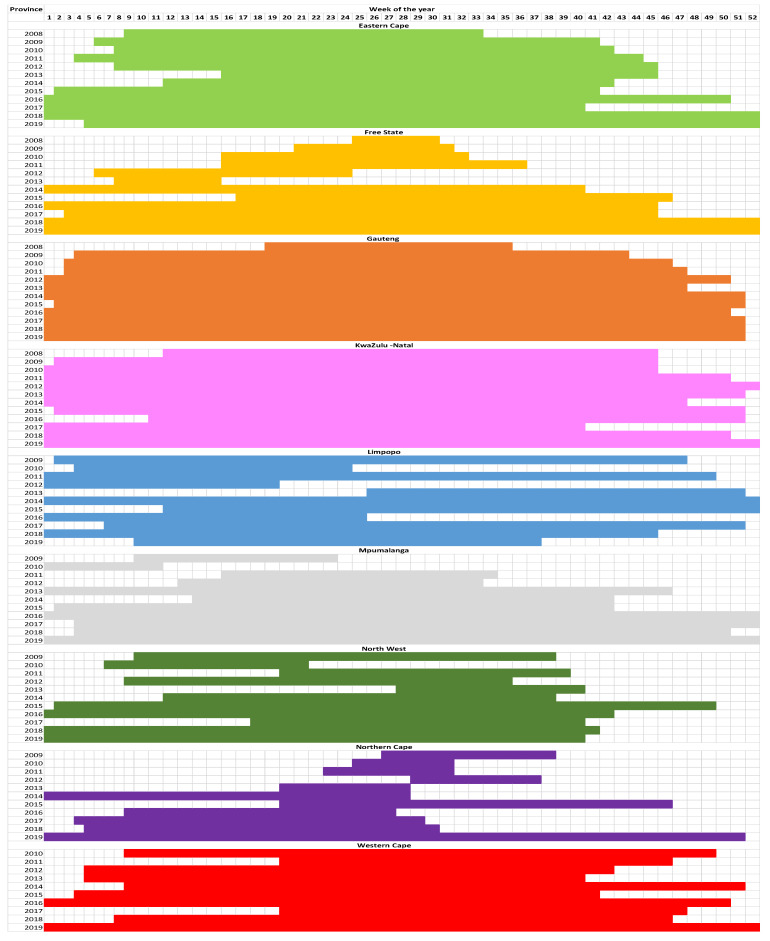
Applying Vega et al.’s [10] 5% threshold for South Africa’s nine provinces.

More consistent with the CDC methodology of intensity thresholds, Vega et al. [10] advocate a 5% threshold. This threshold was applied to the data across all nine provinces from 2008 to 2019, and it is clear that the acute respiratory infectious disease season spans a considerably longer period than 19 weeks (Figure 6). This was particularly the case for provinces that had higher medical insurance members; Eastern Cape, Gauteng, KwaZulu-Natal, and Western Cape well exceeded the 19-week duration from 2008 to 2019 (Figure 6). All of the abovementioned provinces’ cases started as early as the first week and finish in week 51 of the year, meaning that there indeed is no flu season, and South Africans in these province display flu-like symptoms throughout the year (Figure 6). This threshold is also not appropriate for the Free State, Limpopo, Mpumalanga, the Northern Cape, and the North West as in some years the acute lower respiratory disease season is only nine weeks, and in other years it is the entire year and is experienced through warmer seasons and not colder (Figure 6).

**Figure 7:**
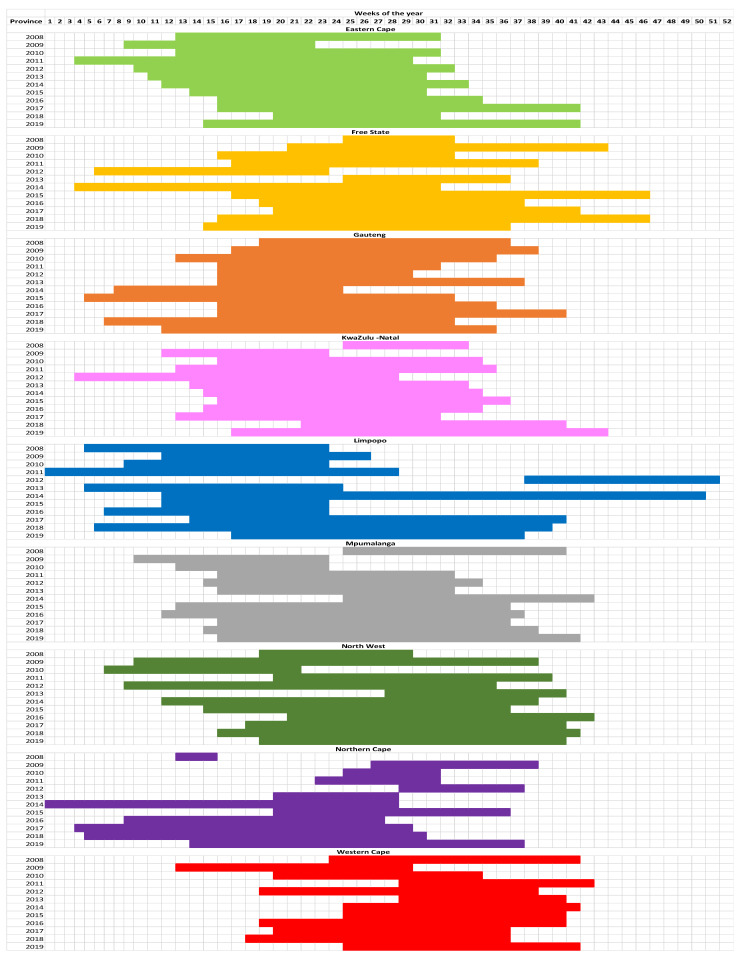
Threshold graphs showing the different thresholds for the nine South African provinces, respectively.

After testing a series of different thresholds for each of the provinces, the most appropriate threshold was determined for each province, respectively, that yielded results that were close to a 19-week flu season from 2008 to 2019: Eastern Cape (17%), Free State (15%), Gauteng (20%), KwaZulu-Natal (25%), Limpopo (15%), Mpumalanga (15%), North West (13%), Northern Cape (11%), and Western Cape (18%) (Figure 7). The provinces had a distinct seasonal pattern. None of the provinces yielded precisely nineteen weeks, but the threshold selected for each province was close to the nineteen-week season proposed by the NICD. Gauteng, Western Cape, KwaZulu-Natal, and Eastern Cape had the most suitable thresholds. All three provinces have acute respiratory infectious disease seasons that start in the winter season (Figure 7). The Northern Cape, Limpopo, Mpumalanga, and Free State yielded seasons that start very early in the year for certain years.

The timing of seasons differed interannually for each season per province, and when comparing provinces, the start and finish times also differed. Limpopo’s season was distinct from other provinces as not only were overall seasons shorter than nineteen weeks, but the season also commenced as early as week 1 in some years, only started in week 38 in 2012 and managed to last almost the entire year in 2014 (Figure 7). In the Free State, the respiratory disease season was exceptionally short in 2008 and 2013 and started earlier in 2014 (Figure 7). The Northern Cape also had some unusual start and finish dates, particularly for 2008 and 2014 (Figure 7). In 2008, Mpumalanga had a respiratory disease season that commenced much later in the year (Figure 7). Gauteng, KwaZulu-Natal, Eastern Cape, and Western Cape on average had respiratory disease seasons that started early in some years but ran through the whole winter season and ended between weeks 37 and 46 (Figure 7).

## 4. Discussion

Annual acute respiratory infectious disease epidemics result in an estimated three to five million cases of severe illness, and about 290,000–650,000 deaths globally [6]. In South Africa, influenza (commonly known as flu) kills between 6000 and 11,000 people every year [37]. About half of these deaths are in the elderly, and about 30% are in HIV-infected people [7,37]. The highest rates of hospitalization are in the elderly (65 years and older), HIV-infected people, and children less than 5 years of age [37]. Pregnant women are also at increased risk of hospitalization and death from flu infections. People with chronic illnesses such as diabetes, lung disease, tuberculosis, and heart disease are also at increased risk of being hospitalized from the flu. During the flu season in South Africa, about 8–10% of patients hospitalized for pneumonia and 25% of patients with flu-like illness (fever and cough) will test positive for influenza.

Across South Africa, seasonality influences a wide range of activities that contribute largely to the economy; however, there is little consensus on the timing of seasonal boundaries [38,39,40,41,42,43]. This is due to the inconsistencies in different ad hoc approaches to defining seasonal boundaries across South Africa [44]. The NICD [7] states that for the past thirteen years, the South African flu season has lasted for approximately 19 weeks. Using the CDC’s approach of intensity thresholds methodology [17], different thresholds had to be applied to the Discovery medical insurance claim data to quantify the South African acute respiratory infectious disease season. Based on the above findings, it is clear that the acute respiratory disease season is most intense during the winter season. Each province had different thresholds; however, all of them increased in the fall and winter based on Van der Walt and Fitchett’s [44] seasons classification. These thresholds vary significantly across provinces. The most appropriate threshold was determined for each province that yielded results that were close to a 19-week flu season: Eastern Cape (17%), Free State (15%), Gauteng (20%), KwaZulu-Natal (25%), Limpopo (15%), Mpumalanga (15%), North West (13%), Northern Cape (11%), and Western Cape (18%) (Figure 7). This could be due to several various reasons: (1) the number of medical insurance members, certain provinces are considered to have more affluent residents who can afford medical insurance, whilst others cannot; (2) the geographical location of the province can contribute to the seasonality of acute respiratory infectious diseases.

A disease’s potential to cause an outbreak determines whether it should be under surveillance [45]. An epidemic threshold indicates the level of incidence above which a disease requires an urgent response [18,45]. Each disease has a specific threshold that depends on its infectiousness, other determinants of transmission, and the degree to which it is locally endemic. Surveillance is often focused on diseases that could cause an outbreak and be a major public health concern. However, there is value in monitoring case numbers in less severe diseases. This allows for better communication with the public and the preparation of health facilities [46]. While it would be very costly to have high-resolution surveillance of the type instituted for COVID-19 where people were extensively tested, medical insurance data provide a proxy by which this can be carried out [47,48]. These thresholds can be evaluated weekly, and when approaching them, the public can be alerted [48]. The NICD already sends out warnings for flu seasons, but this could expand the process at a relatively low cost.

While this study has identified the thresholds that align with the NICD nineteen-week flu season and provides the mean timing of the season, it does not explore the causes of this seasonal pattern. Adequate thresholds will allow for future research to look at other drivers of seasonal trends in acute respiratory infectious disease cases. Cases and claims increase during the winter season which also coincides with school holidays, a time when people migrate, and cluster in spaces, such as airports and tourist destinations, where diseases spread easily. Therefore, it is important for future studies to look at the causal factors.

## 5. Conclusions

In a developing country such as South Africa, pivotal facets of primary healthcare are not in place, and there is a substantial human resources crisis facing the healthcare field [49]. Climatic changes will exacerbate the existing problems experienced by the public healthcare sector in the country [14]. The respiratory disease season is predominantly in the winter season and ends in summer for all nine provinces in South Africa. Each province has a different threshold that yields a respiratory disease season that is roughly 19 weeks long, Eastern Cape (17%), Free State (15%), Gauteng (20%), KwaZulu-Natal (25%), Limpopo (15%), Mpumalanga (15%), North West (13%), Northern Cape (11%), and Western Cape (18%).

This study provided insight into the complexities of varying quantification of the acute respiratory infectious disease season in South Africa, and although the NICD has been able to determine the duration of the influenza season, the start and finish date of the influenza season needs to be determined to better plan for seasonal acute respiratory disease epidemics. Thus, advocating for a particular classification of the acute respiratory disease season is important. This may be beneficial to the public as they can better understand their risk of disease and are subsequently able to mitigate it accordingly and get vaccinated on time. This has the potential to lessen the disease load and risk to human health. It is also of benefit to the medical insurance schemes in planning for surges in claims, thus enhancing their financial sustainability.

## Data Availability

The authors do not have authority to share the data. Requests for access to the date should be made directly to Discovery Health Medical Aid and to the South African Medical Research Council Respiratory and Meningeal Pathogens Research Unit.

## References

[1] Chumakov M.P., Voroshilova M.K., Antsupova A.S., Boiko V.M., Blinova M.I., Priimiagi L.S., Rodin V.I., Seibil V.B., Siniak K.M., Smorodintsev A.A. (1992). Live enteroviral vaccines for the emergency nonspecific prevention of mass respiratory diseases during fall-winter epidemics of influenza and acute respiratory diseases. Zhurnal Mikrobiol. Epidemiol. I Immunobiol..

[2] Cooper R.J., Hoffman J.R., Bartlett J.G., Besser R.E., Gonzales R., Hickner J.M., Sande M.A., American Academy of Family Physicians, American College of Physicians-American Society of Internal Medicine, Centres for Disease Control (2001). Principles of appropriate antibiotic use for acute pharyngitis in adults: Background. Ann. Intern. Med..

[3] Fendrick A.M., Monto A.S., Nightengale B., Sarnes M. (2003). The economic burden of non-influenza-related viral respiratory tract infection in the United States. Arch. Intern. Med..

[4] Nicholson S.E. (2000). The nature of rainfall variability over Africa on time scales of decades to millenia. Glob. Planet. Chang..

[5] Molinari N.A., Ortega-Sanchez I.R., Messonnier M.L., Thompson W.W., Wortley P.M., Weintraub E., Bridges C.B. (2007). The annual impact of seasonal influenza in the US: Measuring disease burden and costs. Vaccine.

[6] WHO (2012). Vaccines against influenza WHO position paper—November 2012. Wkly. Epidemiol. Rec..

[7] NICD. https://www.nicd.ac.za/flu-season-is-around-the-corner/#:~:text=Although%20the%20timing%20of%20the,years%20has%20been%2019%20weeks.

[8] Serfling R.E. (1963). Methods for current statistical analysis of excess pneumonia-influenza deaths. Public Health Rep..

[9] Cowling B.J., Wong I.O.L., Ho L.M., Riley S., Leung G.M. (2006). Methods for monitoring influenza surveillance data. Int. J. Epidemiol..

[10] Vega T., Lozano J.E., Meerhoff T., Snacken R., Beauté J., Jorgensen P., Ortiz de Lejarazu R., Domegan L., Mossong J., Nielsen J. (2015). Influenza surveillance in Europe: Comparing intensity levels calculated using the moving epidemic method. Influenza Other Respir. Viruses.

[11] Meerhoff T.J., Paget W.J., Aguilera J.F., van der Velden J. (2004). Harmonizing the virological surveillance of influenza in Europe: Results of an 18-country survey. Virus Res..

[12] Wang J., Guan Y., Wu L., Guan X., Cai W., Huang J., Dong W., Zhang B. (2021). Changing Lengths of the Four Seasons by Global Warming. Geophys. Res. Lett..

[13] Smit A.J., Fitchett J.M., Engelbrecht F.A., Scholes R.J., Dzhivhuho G., Sweijd A.N. (2020). Winter Is Coming: A Southern Hemisphere Perspective of the Environmental Drivers of SARS-CoV-2 and the Potential Seasonality of COVID-19. Int. J. Res. Public Health.

[14] Wright C.Y., Kapwata T., Jean du Preez D., Wernecke B., Garland R.M., Nkosi V., Landman W.A., Dyson L., Norval M. (2021). Major climate change-induced risks to human health in South Africa. Environ. Res..

[15] Cannell J.J., Vieth R., Umhau J., Holick M., Grant W., Madronich S., Garland C., Giovannucci E. (2006). Epidemic influenza and vitamin D. Epidemiol. Infect..

[16] Huang D., Taha M.S., Norcera A.L., Workman M.D., Amiji M.M., Bleier B.S. (2022). Cold exposure impairs extracellular vesicle swarm-mediated nasal antiviral immunity. J. Allergy Clin. Immunol..

[17] CDC. https://www.cdc.gov/flu/about/season/flu-season.htm.

[18] Biggerstaff M., Kniss K., Jernigan D.B., Brammer L., Bresee J., Garg S., Burns E., Reed C. (2018). Systematic Assessment of Multiple Routine and Near Real-Time Indicators to Classify the Severity of Influenza Seasons and Pandemics in the United States, 2003–2004 through 2015–2016. Am. J. Epidemiol..

[19] Petrie J.G., Lauring A.S., Martin E.T., Kaye K.S. (2020). Hospital Associated Respiratory Virus Infection in Children and Adults: It Does Not Just Occur During Cold and Flu Season. Open Forum Infect. Dis..

[20] Eurich D.T., Marrie T.J., Johnstone J., Majumdar S.R. (2008). Mortality Reduction with Influenza Vaccine in Patients with Pneumonia Outside “Flu” Season Pleiotropic Benefits or Residual Confounding?. Am. J. Respir. Crit. Care Med..

[21] Landman W.A., Malherbe J., Engelbrecht F., Mambo J., Faccer K. (2017). South Africa’s present-day climate. Understanding the Social and Environmental Implications of Global Change.

[22] Lennard C., Knight J., Rogerson C.M. (2019). Multi-Scale Drivers of the South African Weather and Climate. The Geography of South Africa.

[23] Botai C.M., Botai J.O., Adeola A.M. (2018). The spatial distribution of temporal precipitation contrasts in South Africa. S. Afr. J. Sci..

[24] Lennard C., Hegerl G. (2015). Relating changes in synoptic circulation to the surface rainfall response using self-organising maps. Clim. Dyn..

[25] Schulze R.E., Maharaj M., Schulze R.E. (2007). Rainfall seasonality. South African Atlas of Climatology and Agrohydrology.

[26] Roffe S.J., Fitchett J.M., Curtis C.J. (2019). Classifying and mapping rainfall seasonality in South Africa: A review. S. Afr. Geogr. J..

[27] Taljaard J.J. (1996). Atmospheric Circulation Systems, Synoptic Climatology, and Weather Phenomena of South Africa. Part 6. Rainfall in South Africa.

[28] Tyson P.D., Preston-Whyte R.A. (2000). The Weather and Climate of Southern Africa.

[29] Jury M.R. (2018). Climate trends across South Africa since 1980. Water SA.

[30] Maphumulo W.T., Bhengu B.R. (2019). Challenges of quality improvement in the healthcare of South Africa post-apartheid: A critical review. Curations.

[31] Republic of South Africa Department of Health (2015). National Health Insurance for South Africa: Towards Universal Health Coverage.

[32] Mail & Guardian. http://mg.co.za/article/2015-08-22-nhi-to-reduce-cost-of-healthcare.

[33] NHI White Paper Released. http://www.sanews.gov.za/south-africa/nhi-white-paper-released.

[34] Suliman R., Mtsweni J. (2022). Adding up the numbers: COVID-19 in South Africa. S. Afr. J. Sci..

[35] Horwitz S. (2013). Baragwanath Hospital, Soweto: A History of Medical Care 1941–1990.

[36] Huddle K.R.L. (2013). Baragwanath Hospital, Soweto: A History of Medical Care 1941–1990: A Short Review.

[37] NICD. https://www.nicd.ac.za/influenza-season-approaching/.

[38] Battisti D.S., Naylor R.L. (2009). Historical warnings of future food insecurity with unprecedented seasonal heat. Science.

[39] Tshiala M.F., Olwoch J.M., Engelbrecht F.A. (2011). Analysis of temperature trends over Limpopo Province, South Africa. J. Geogr. Geol..

[40] De Cian E., Lanzi E., Roson R. (2013). Seasonal temperature variations, and energy demand. Clim. Chang..

[41] Fitchett J., Robinson D., Hoogendoorn G. (2016). Climate suitability for tourism in South Africa. J. Sustain. Tour..

[42] Lazenby M.J., Landman W.A., Garland R.M., DeWitt D.G. (2014). Seasonal temperature prediction skill over southern Africa and human health. Meteorol. Appl..

[43] Ziervogel G., New M., Van Garderen E.A., Midgley G., Taylor A., Hamann R. (2014). Climate change impacts and adaptation in South Africa. Clim. Chang..

[44] Van der Walt A.J., Fitchett J.M. (2020). Statistical classification of South African seasonal divisions based on daily temperature data. S. Afr. J. Sci..

[45] UNHCR. https://emergency.unhcr.org/entry/115610/disease-surveillance-thresholds.

[46] Parham P.E., Michael E. (2010). Modeling the Effects of Weather and Climate Change on Malaria Transmissions. Environ. Health Perspect..

[47] Tempia S., Walaza S., Bhiman J.N., McMorrow M.L., Moyes J., Mkhencele T., Meiring S., Quan V., Bishop K., McAnerney J.M. (2021). The decline of influenza and respiratory syncytial virus detection in facility-based surveillance during the COVID-19 pandemic, South Africa, January to October 2020. Eurosurveillance.

[48] Jassat W., Karim S.S.A., Mudara C., Welch R., Ozougwu L., Groome M.J., Govender N., von Gottberg A., Wolter N., Wolmarans M. (2022). Clinical severity of COVID-19 in patients admitted to hospital during the omicron wave in South Africa: A retrospective observational study. Lancet.

[49] Albuquerque E. (2019). Brazil and the middle-income trap: Its historical roots. Seoul J. Econ..

